# Late Diagnosis of Anomalous Aortic Origin of a Coronary Artery from the Inappropriate Sinus of Valsalva during Investigation of Chest Pain

**DOI:** 10.1155/2018/3879243

**Published:** 2018-01-30

**Authors:** Brunna Priscylla Américo Carvalho, Marcio Antônio Dos Santos, Wilson Pedro Guimarães Neto, Júlio César Queiroz De França, Márcio Rogério de Souza Braite, Rodrigo Bottura De Araújo, Isabella Gomes Carvalho, Moacir Fernandes de Godoy

**Affiliations:** ^1^Cardiology and Cardiovascular Surgery Department, São José do Rio Preto Medical School, São José do Rio Preto, SP, Brazil; ^2^Hemodynamic and Interventional Cardiology Service, Hospital de Base, São José do Rio Preto, SP, Brazil

## Abstract

In this work are reported two cases of anomalous aortic origin of a coronary artery (AAOCA), with the left main coronary artery (LMCA) arising at the right sinus of Valsalva in a 77-year-old woman and in a 79-year-old man submitted to angiography after positive ischemic tests. The origin of the LMCA or the left descendant artery (LDA) from the right sinus of Valsalva has a prevalence of 0.2%, the origin of the circumflex artery (CXA) from the right sinus 0.5%, and the origin of the right coronary artery (RCA) from the left sinus of Valsalva has a prevalence of 0.3%. It is the subgroup of the coronary anomalies that has the greatest potential for clinical repercussions, especially the sudden cardiac death (SCD). We discuss the diagnostic methods and treatment options for this kind of coronary anomaly in symptomatic cases.

## 1. Introduction

The anomalous aortic origin of a coronary artery (AAOCA) is a rare disease with a range in prevalence from 0.3% to 1.5%, and it can be fatal depending on its anatomy [1, 2]. The symptoms may appear in childhood or only in adult life when the diagnosis is, most of the times, late due to normal ischemic tests. With the advancement of image methods, we may have a better understanding of the coronary anatomy and an early confirmation of this anatomical variant.

The objective of this report is to show the image diagnosis of two cases of anatomical variation of the left main coronary artery that arises at the right semilunar leaflet of the aortic valve in patients evaluated in the emergency room for precordial pain and treatment options for the symptomatic cases.

## 2. Case Reports

### 2.1. Case 1

**Figure 1:**
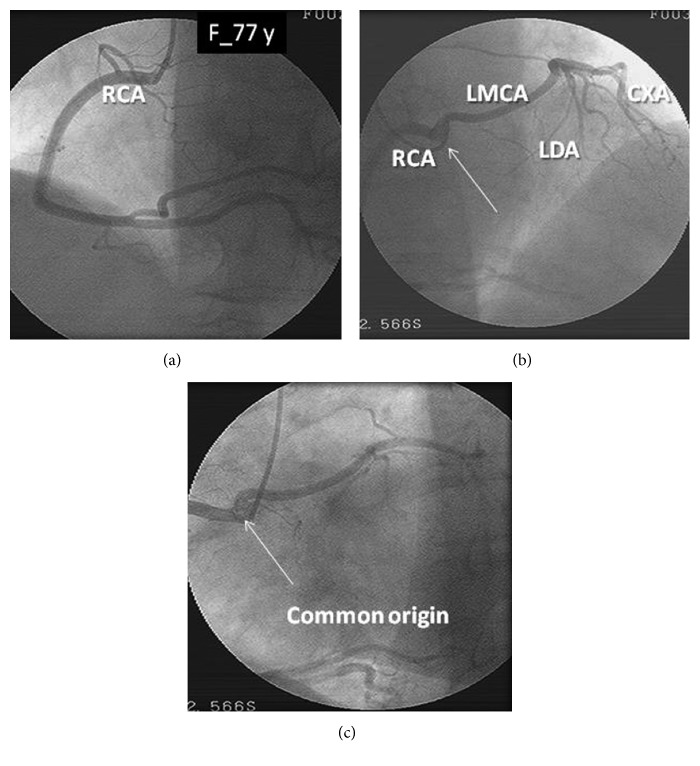
Coronary angiography showing the right coronary artery and the left main coronary artery arising from the same ostium, with a prepulmonic course of the LMCA.

A 77-year-old woman, with high blood pressure, in use of atenolol and losartan, presented to the emergency room with a complaint of chest pain that began during rest, tightness-like, without irradiation, of great intensity, associated with nausea, becoming worse during inspiration, and getting better at rest. The initial electrocardiogram showed atrial fibrillation, and the dosages of troponins were negative. A scintigraphy was performed with sestamibi that showed myocardial ischemia of small-to-moderate extension in the anteroseptal and inferoapical regions of the left ventricle (LV). Invasive stratification performed with coronary angiography showed an anomalous aortic origin of the left main coronary artery at the right semilunar cusp of the aortic valve, with a prepulmonic course and sharing a common ostium with the RCA (Figure 1). There were no significant coronary obstructions. Synchronized scintigraphy of cardiac chambers showed enlarged left ventricle with global contractile performance depressed in mild-to-moderate grades and a left ventricular ejection fraction (LVEF) of 0.36. As the coronary anomaly was a fortuitous discovery and not related to the myocardial perfusion imaging abnormalities, the patient was discharged for clinical treatment using furosemide 40 mg once a day, carvedilol 25 mg twice a day, losartan 50 mg twice a day, and simvastatin 40 mg per day.

### 2.2. Case 2

**Figure 2:**
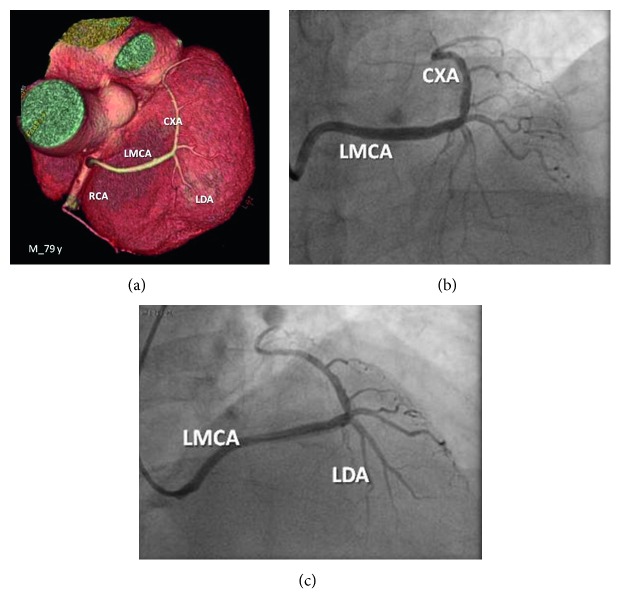
CT angiography and coronary angiography showing a long and trifurcated left main coronary artery with a prepulmonic course.

A 79-year-old man, farmer, with no history of medication intake, presented to the emergency room with chest pain, started at physical stress, tightness-like, of moderate intensity, without irradiation, without associated factors, and getting better at rest. The electrocardiogram showed sinus rhythm, without suggestive signals of myocardial ischemia, and dosages of troponins were negative. The scintigraphy with sestamibi showed myocardial ischemia of a small-to-moderate area, involving parts of the apical region and inferoapical and inferior regions of the left ventricle. Invasive stratification performed with coronary angiography showed the anomalous aortic origin of the left main coronary artery at the right semilunar cusp of the aortic valve. The CT angiography showed a long and trifurcated left main coronary artery, with a prepulmonic course (Figure 2). Once again, this AAOCA pattern was not recognized as potentially serious, and the patient was discharged asymptomatic.

## 3. Discussion

Coronary anomaly can be defined as any change in the anatomical pattern, such as the number of ostium, proximal course, distal bed, and it is rarely found in the general population [3].

The anomalous aortic origin of the left main coronary artery from the right aortic sinus may be related to SCD, usually during or after exhausting exercise. It is currently the second most common cause of SCD in athletes [2]. The anatomical variation can follow five different paths: interarterial, subpulmonic (intraconal or intraseptal), prepulmonic, retroaortic, or retrocardiac. The interarterial variation is the pattern that has stronger relationship with SCD [2, 4, 5].

In most cases of AAOCA, there are no symptoms, and the only clinical manifestation of the interarterial course may be SCD. When symptomatic, the patients may complain of syncope, chest pain, and palpitations. The lack of symptoms difficults the diagnosis. In symptomatic patients, the initial investigation should follow with electrocardiogram, stress testing, and echocardiography [5].

The coronary angiography is traditionally the gold standard for the diagnosis of these anomalies, with the disadvantage of being an invasive test that uses potentially nephrotoxic contrast and ionizing radiation. Besides that, the usual two-dimensional images may generate failures in interpretation [6].

On the other hand, computed tomography of coronary arteries has had a fast evolution as the coronary evaluation method. This method has shown excellent accuracy in evaluating the origin and proximal course of the coronary anomalies, being superior to coronary angiography. The middle and distal segments have also been better visualized. Some comparative studies with coronary angiography have shown superior efficiency, suggesting that multislice computed tomography is the ideal test for coronary anomaly diagnosis [3, 6].

Transthoracic echocardiogram is the most useful method for children because it is not invasive and does not use radiation, contrast, or sedation. It also allows visualization of the origin of the coronary vessels [7].

In asymptomatic patients, there is no consensus on treatment management. However, in symptomatic individuals, with interarterial course, surgical treatment should be the first option, but there are no data that define which is the best surgical technique to be used. Coronary artery bypass grafting should be avoided, unless there is concomitant obstructive coronary artery disease [4]. Surgical techniques are beyond the scope of this work and will not be discussed. In some symptomatic individuals or in cases with reversible myocardial ischemia, beta-blockers and calcium channel blockers can be used in order to reduce ischemic symptoms [6, 7].

## 4. Conclusion

The two cases presented above show patients with suspected acute coronary artery syndromes, in which the clinical investigation with invasive strategy diagnosed anomalous aortic origin of the left main coronary artery without atherosclerotic obstructions and no relationship with the myocardial ischemic changes. These case reports highlight this rare diagnostic possibility in patients undergoing angiography.
